# Lambdoid Suture Defect in a 12-year-old Neurofibromatosis Patient

**DOI:** 10.7759/cureus.54567

**Published:** 2024-02-20

**Authors:** Hend Almahmood, Sarah Al-Sayed, Wahid Agab

**Affiliations:** 1 Pediatrics, Bahrain Defense Force Hospital, Riffa, BHR

**Keywords:** café-au-lait macules, plexiform neurofibromas, skull defect, lambdoid suture defect, neurofibromatosis type-1

## Abstract

Neurofibromatosis type 1 (NF-1) is the most common neurocutaneous syndrome. It is inherited in an autosomal dominant manner, with many patients having the syndrome as the result of a de novo mutation. NF-1 is caused by a mutation in the NF-1 gene located on the chromosome 17q11.2. NF-1 gene mutations result in the absence or reduced function of neurofibromin protein, thereby promoting tumor development and other clinical findings. NF-1 is fully penetrant, and it is commonly manifested by café-au-lait macules, axillary and/or inguinal freckling, neurofibromas, and Lisch nodules in the eyes. Skeletal manifestations include scoliosis, short stature, long bone dysplasia, and pseudoarthrosis. Rarely, NF-1 can manifest lambdoid suture defects. This report describes the case of a 12-year-old neurofibromatosis patient who presented to the pediatric clinic with a palpable posterior scalp defect, as well as café-au-lait macules and Lisch nodules. Diagnosis of NF-1 was made clinically. MRI and CT scan were done, and the patient was diagnosed with a lambdoid suture defect that is not associated with plexiform neurofibroma. Moreover, whole exome sequence (WES) was done, and diagnosis of NF-1 was confirmed. Watchful waiting and continuous monitoring were the management of choice for this case.

## Introduction

Neurofibromatosis type 1 (NF-1), previously known as Von Recklinghausen disease, is the most common neurocutaneous syndrome. It is an autosomal dominant inherited syndrome, with 50% of the patients having NF-1 as the result of a de novo mutation [1]. NF-1 is caused by a mutation in the NF-1 gene located on the chromosome 17q11.2. NF-1 gene encodes for a protein called neurofibromin, which is an inhibitor and regulator of the oncogene RAS. Mutations and loss of production of neurofibromin will lead to overactivation of RAS, thereby promoting tumor development [2].

Although clinical manifestations of NF-1 are highly variable [3], all generations carrying the mutation are affected, as the disease is fully penetrant. Moreover, NF-1 affects approximately 1:2600 to 1:3000 individuals [4], and it is characterized by cafe-au-lait macules, axillary and/or inguinal freckling, neurofibromas, and Lisch nodules in the eye. Skeletal manifestations of NF-1 have been noted in approximately 50% of the patients [5], these manifestations include scoliosis, short stature, long bone dysplasia, and pseudoarthrosis. A distinguishing osseous lesion such as sphenoid dysplasia, pseudarthrosis, or anterolateral bowing of the tibia is one of the diagnostic criteria for NF-1.

Out of those skeletal abnormalities, skull bone defects are of unusual occurrence, especially lambdoid suture defects. The majority of the reported lambdoid suture defects are associated with underlying or overlying plexiform neurofibromas that lead to progressive bone resorption. In this report, we present a case of NF-1 with a lambdoid suture defect that is not attributed to plexiform neurofibroma. A very limited number of similar cases have been reported and published before [6,7].

## Case presentation

**Figure 1:**
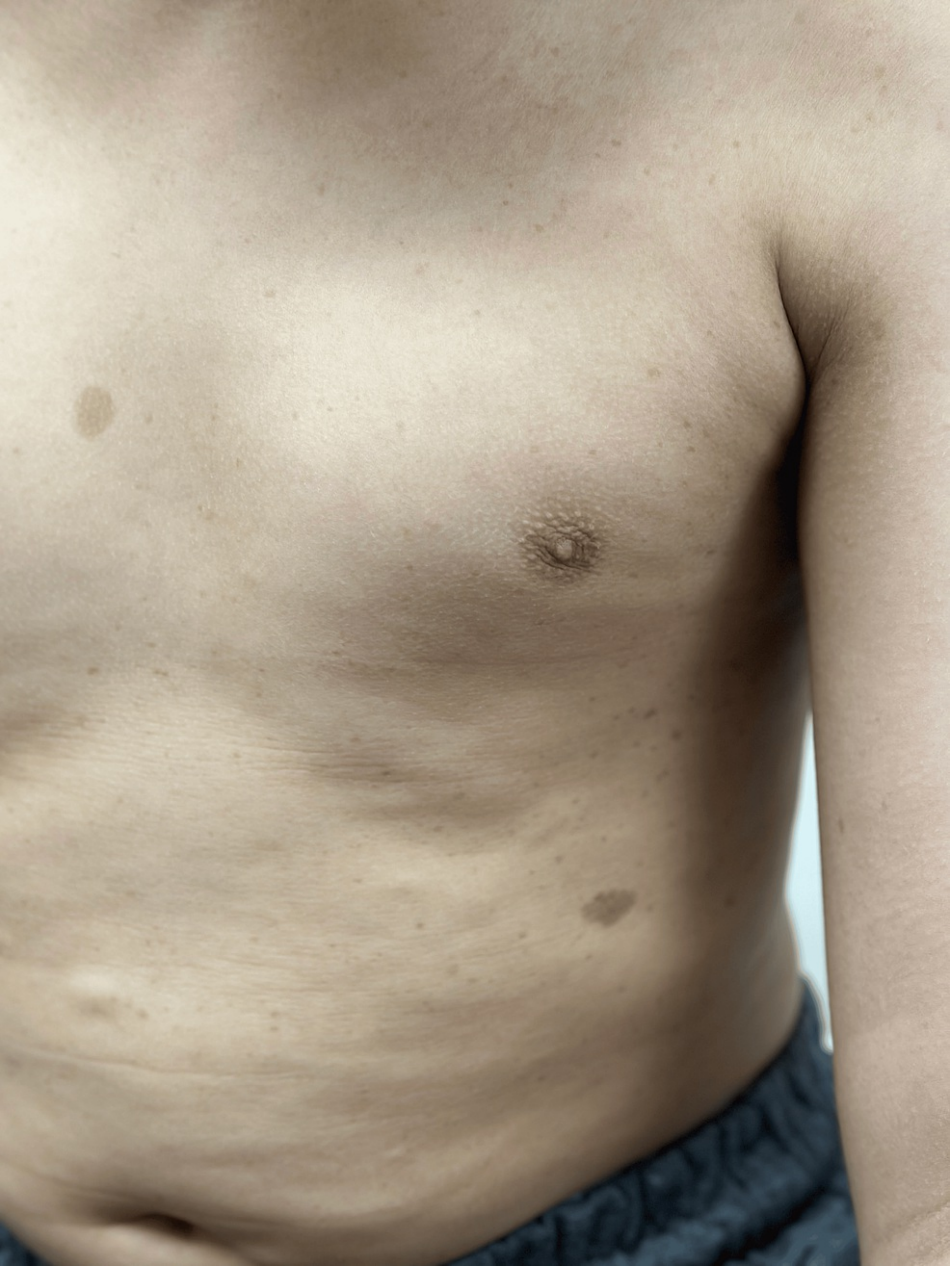
Café-au-lait macules

**Figure 2:**
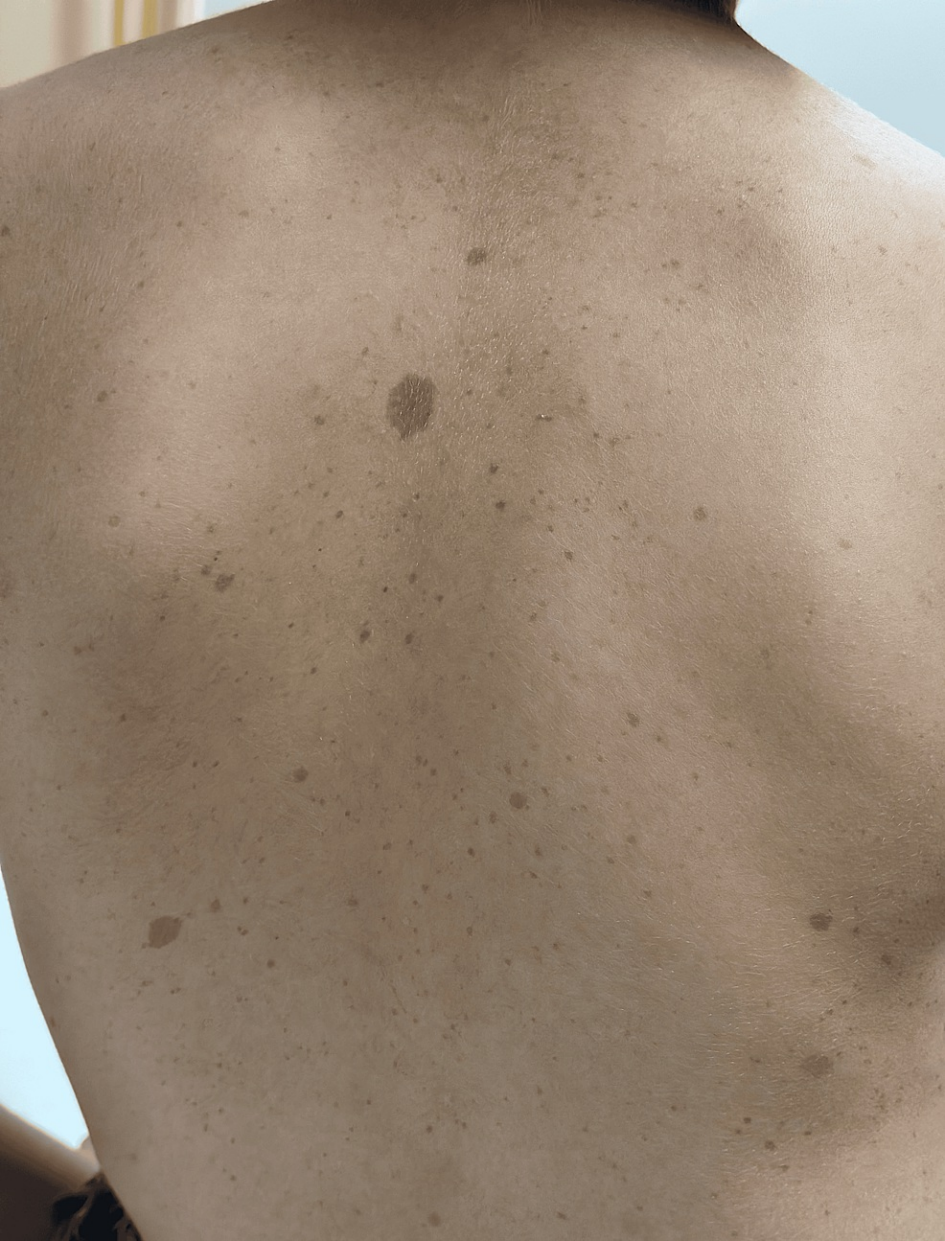
Café-au-lait macules

**Figure 3:**
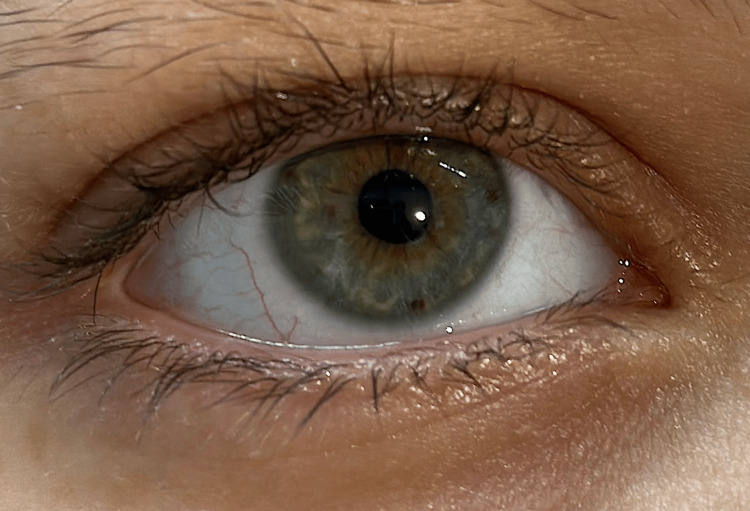
Lisch nodules

A boy aged 12 years and eight months presented to the pediatric clinic with the complaint of a palpable posterior scalp defect. Upon examination, the patient had multiple café-au-lait macules with the size of >15 mm in greatest diameter, distributed over the trunk and thighs (Figures 1, 2), Lisch nodules in both eyes (Figure 3), and a palpable asymptomatic occipital skull defect. However, there were not any skin freckling or cutaneous neurofibromas. The patient also had short stature, with a height of 139 cm, growing on the third centile. His mid-parental height was 172 cm. Neurological examination was otherwise normal.

The patient fulfilled the criteria of NF-1 and he was officially clinically diagnosed at the time. The diagnosis was later confirmed by genetic testing. According to the father of the patient, they previously noticed the café-au-lait macules; however, they did not seek any medical explanation or diagnosis. There is a positive family history of multiple café-au-lait macules in his mother, his four brothers, his sister, and his two maternal aunts. They were all also clinically diagnosed with NF-1 at the same time and their diagnosis is not yet confirmed by genetic testing. Nonetheless, the father is healthy. 

**Figure 4:**
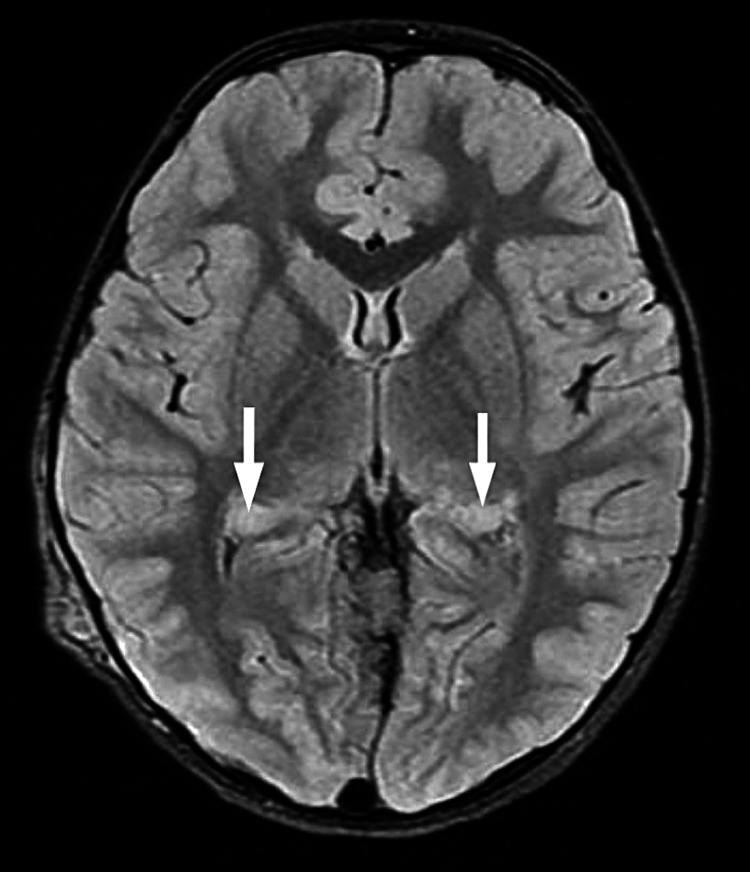
NF-associated bright spots

**Figure 5:**
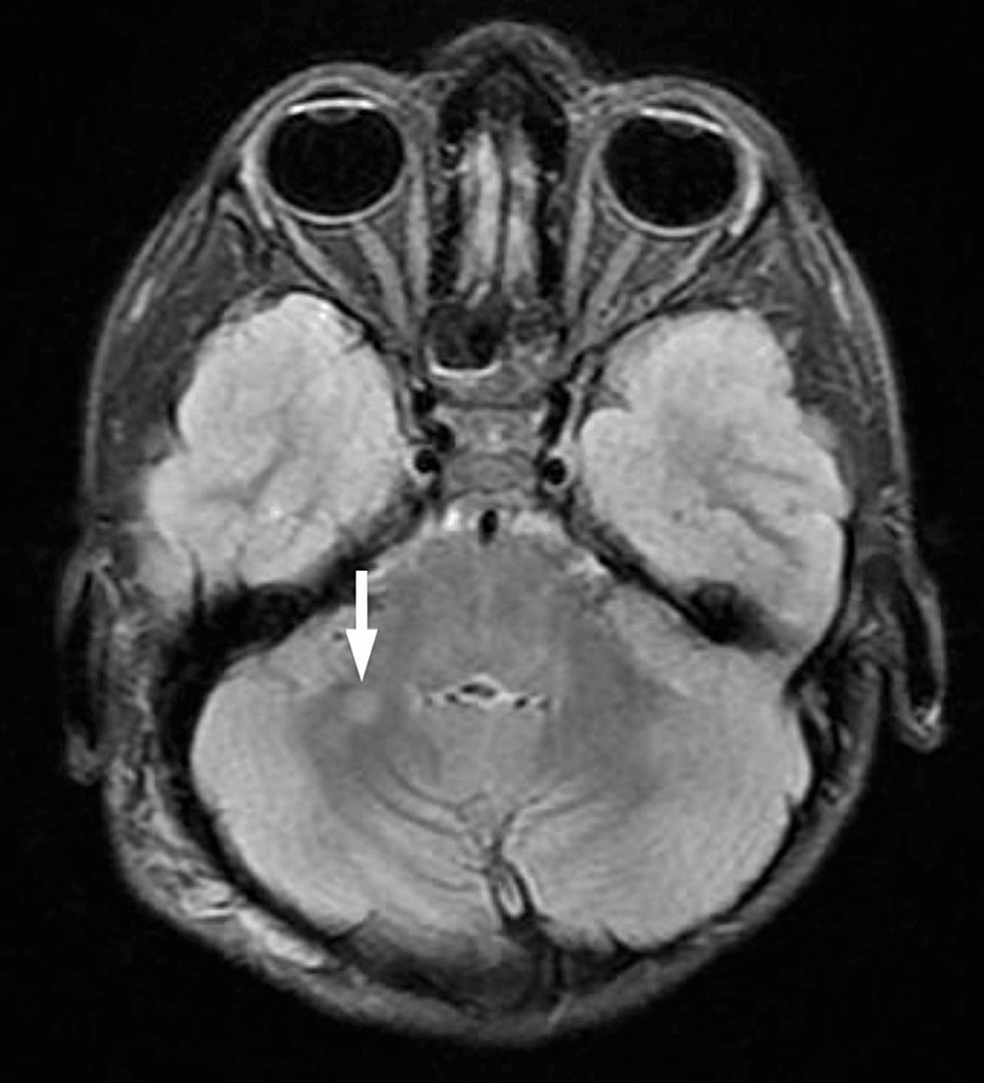
NF-associated bright spots

Magnetic resonance imaging (MRI) was done, and findings were as follows: altered signal intensity focus bright on the T2 weighted image (T2WI) and the fluid-attenuated inversion recovery (FLAIR) images, and isointense on the T1WI noted at the globus pallidus on the left and involving the right middle cerebellar peduncle as well. These findings suggest NF-associated bright spots (Figures 4, 5).

**Figure 6:**
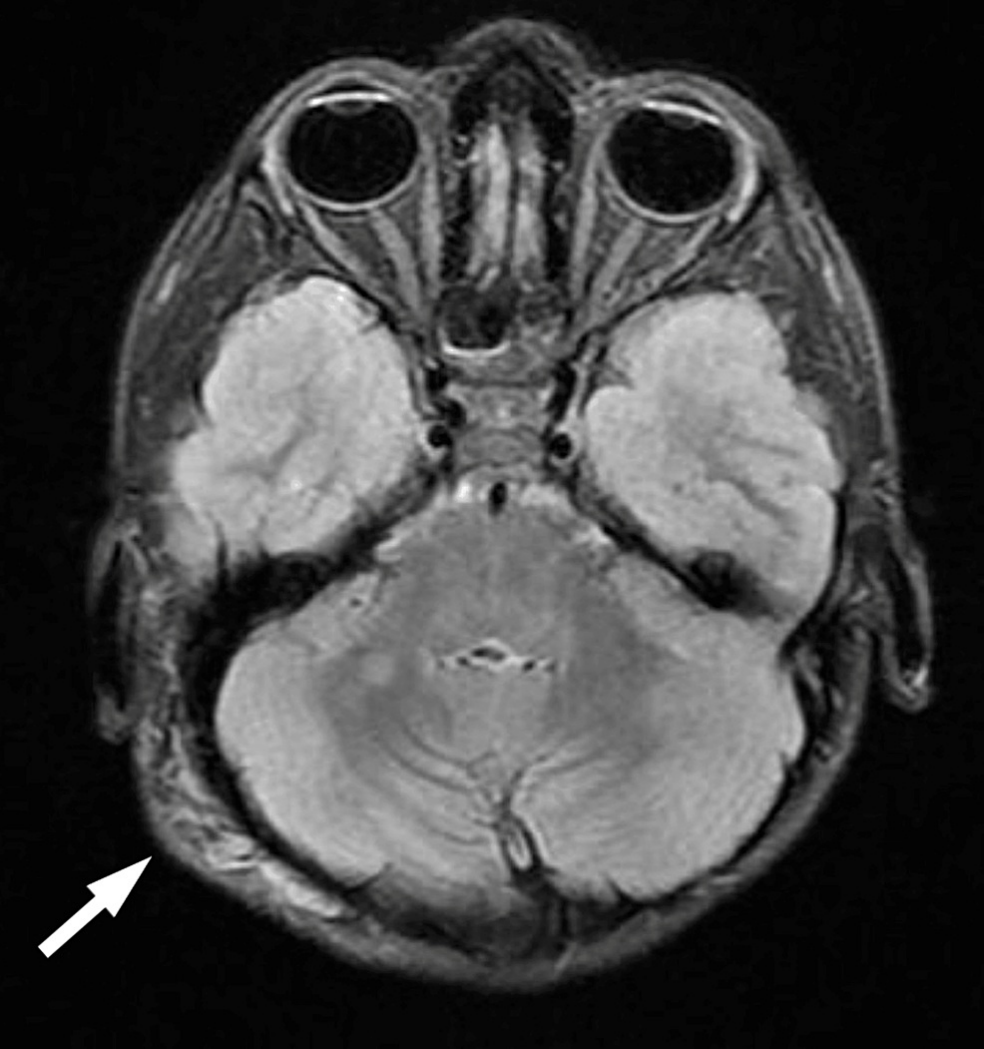
Plexiform neurofibromas

Altered signal-intensity plaque-like infiltrative lesions were noted at the level of the scalp in the parieto-occipital region. This focus demonstrates heterogeneous intensity on the T2WI with central hypointense foci and is isointense on the T1WI, demonstrating post-contrast enhancement. These are likely to represent plexiform neurofibromas (Figure 6). 

**Figure 7:**
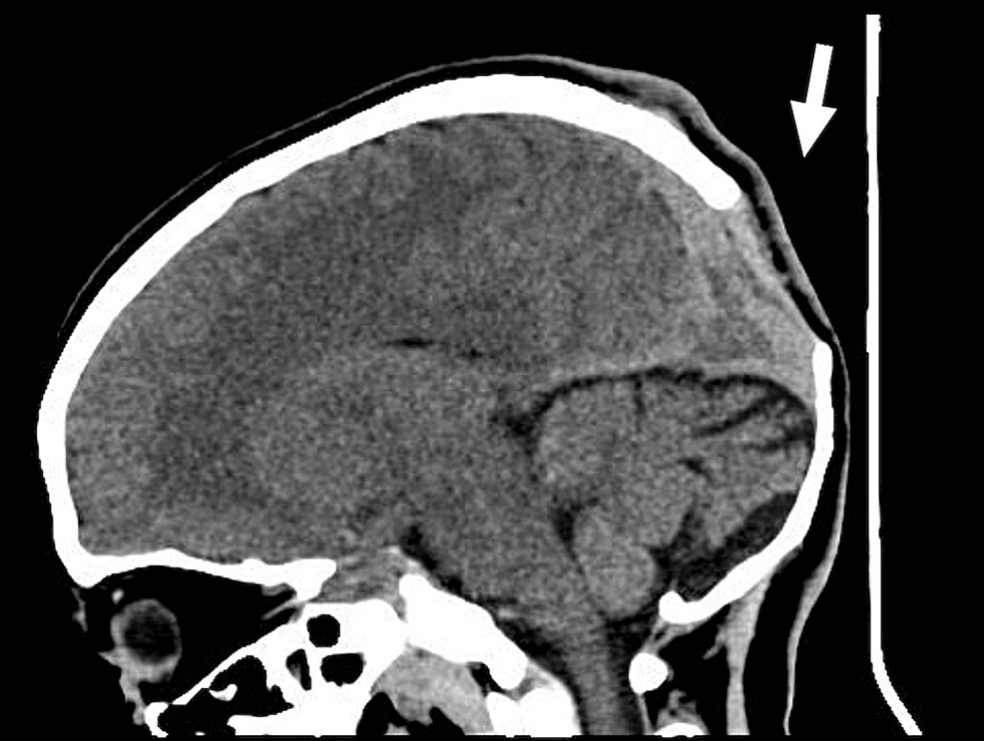
Lambdoid suture bony defect

**Figure 8:**
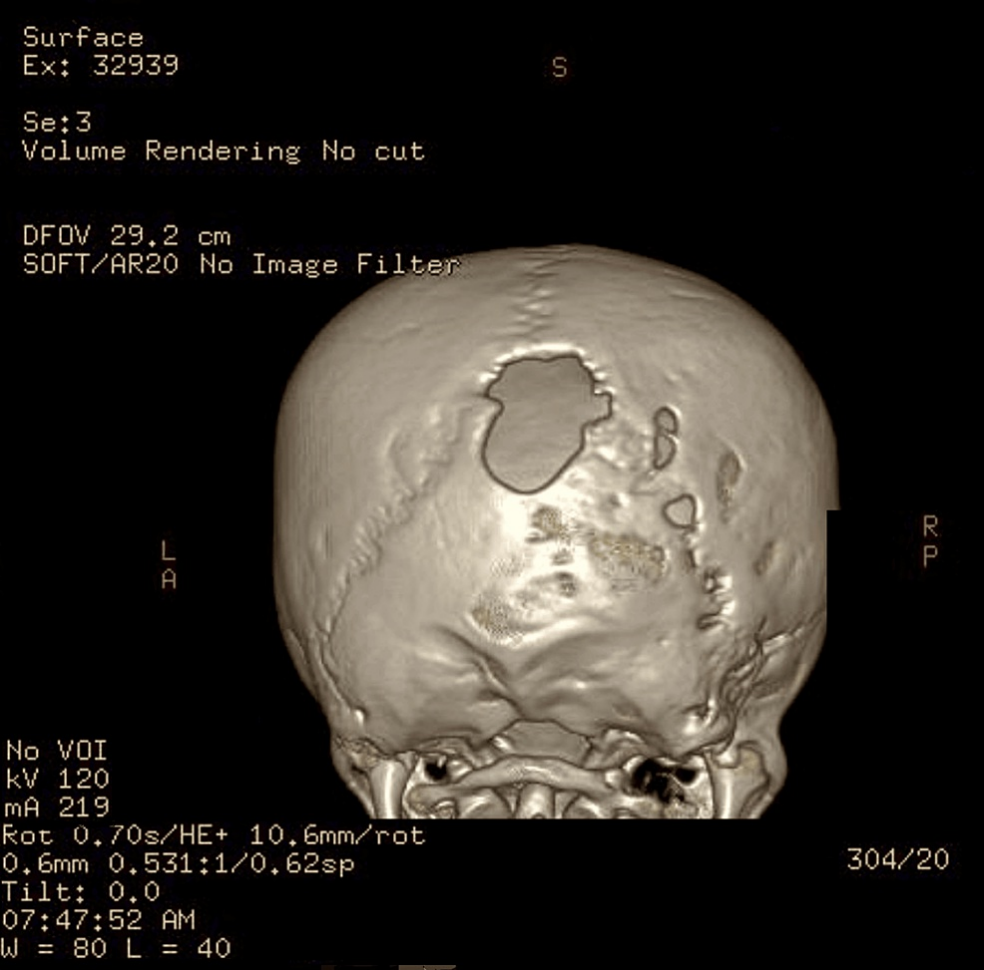
Three-dimensional CT skull showing lambdoid suture bony defect

**Figure 9:**
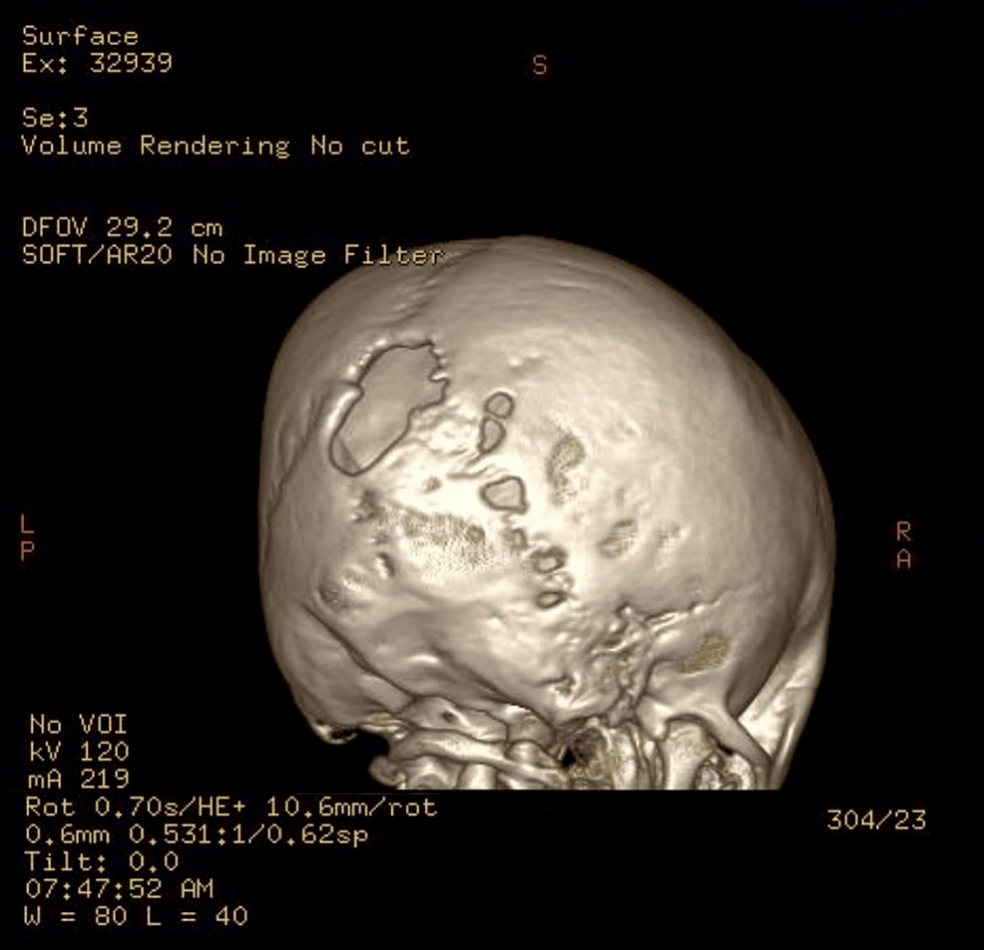
Three-dimensional CT skull showing lambdoid suture bony defect

There were also skull osseous changes on the left side that were confirmed later by doing a brain computed tomography (CT) scan. There were no optic nerve gliomas or focal space-occupying lesions and no lesions in the cord. There was no evidence to suggest tuberous sclerosis. CT brain and three-dimensional (3D) CT skull both showed evidence of lambdoid suture bony defect, as well as other smaller skull defects (Figures 7-9). Nevertheless, no obvious sphenoid wing dysplasia was noted. The ventricular system was normal in size and morphology for the patient’s age. The basal cisterns were normal. The cerebral parenchyma was unremarkable. No midline shift and no obvious optic nerve lesion. 

Whole exome sequence (WES) test was done for the patient, and a heterozygous pathogenic variant was identified in the NF-1 gene, which is consistent with a genetic diagnosis of autosomal dominant NF-1. However, no further clinically relevant variants related to the described phenotype were detected. 

As a secondary finding, a heterozygous pathogenic variant was identified in the BRCA1 gene, and it is consistent with a high risk of developing symptoms of autosomal dominant BRCA1-associated khereditary breast and ovarian cancer (HBOC) syndrome. According to the of the WES test results of this patient, screening of the whole family was suggested.

## Discussion

This patient presented with a palpable occipital bulge that is caused by a significant lambdoid suture defect, along with other smaller parieto-occipital defects. No plexiform neurofibromas were identified underlying the lambdoid defect on the brain MRI or CT scan. Nonetheless, the other smaller skull defects were associated with plexiform neurofibromas seen in brain MRI. 

So far, calvarial bone defects in the region of the lambdoid suture have been reported in very few publications and the majority of them have been attributed to overlying plexiform neurofibromas and/or dural ectasia requiring surgical intervention, and these plexiform neurofibromas can lead to progressive bone resorption. In this report, we presented a case of NF-1 with lambdoid suture bone defect without associated plexiform neurofibromas.

The exact pathophysiology of skull bone defects is still unclear. There are multiple hypotheses that could explain cranial vault defects that are seen in NF-1. Most researchers advocate that NF-1-related bone defects may be acquired and caused by the increased external pressure exerted by underlying plexiform neurofibromas and meningiomas, thus leading to bone corrosion [8,9]. However, one theory suggests that the mutation in the NF-1 gene itself results in intrinsic bone development abnormality related to neurofibromin protein deficiency, as well as defective mesodermal and neuroectodermal development. This theory was suggested to explain cranial lesions with no adjacent masses or tumors [9]. 

Until this day, there are no guidelines for the management of these skull defects in NF-1 patients. Cranial reconstructions were attempted in some patients [9], and it was found that the results were unpredictable and that it has a high possibility for failure because of the continuous bone resorption. For this reason, our patient did not receive any intervention and is being followed up by a neurosurgeon, as watchful waiting and regular follow-up visits are the best management for skull defects, especially in asymptomatic patients. 

## Conclusions

NF-1 is a multisystem inherited syndrome, affecting the skin, bones, soft tissues, and nervous system. Lambdoid suture defects are one of the uncommon skeletal manifestations and complications of NF-1. Most frequently, these defects are related to the exogenous force of plexiform neurofibromas; however, neurofibromin protein deficiency itself can lead to intrinsic bone tissue abnormality, causing defects without adjacent plexiform neurofibromas. Fortunately, the majority of patients with lambdoid suture defects are asymptomatic, and these defects do not usually require surgical intervention. Ongoing follow-up and continuous monitoring are the best choice for management.
